# Development of novel EST‐SSR markers for *Ephedra sinica* (Ephedraceae) by transcriptome database mining

**DOI:** 10.1002/aps3.1212

**Published:** 2019-01-16

**Authors:** Si‐Qian Jiao, Yan‐Qiang Sun, Dong‐Xu Zhang, Qiong Gao, Yuqing Jin, Hui Liu, Yongpeng Ma, Yong Yang, Ilga Porth, Jian‐Feng Mao

**Affiliations:** ^1^ Beijing Advanced Innovation Center for Tree Breeding by Molecular Design National Engineering Laboratory for Tree Breeding Key Laboratory of Genetics and Breeding in Forest Trees and Ornamental Plants Ministry of Education College of Biological Sciences and Technology Beijing Forestry University Beijing 100083 People's Republic of China; ^2^ College of Life Science Datong University Datong 037009 Shanxi People's Republic of China; ^3^ Yunnan Key Laboratory for Integrative Conservation of Plant Species with Extremely Small Populations Germplasm Bank of Wild Species Kunming Institute of Botany Chinese Academy of Sciences Kunming 650201 People's Republic of China; ^4^ State Key Laboratory of Systematic and Evolutionary Botany Institute of Botany Chinese Academy of Sciences Beijing 100093 People's Republic of China; ^5^ Institute for System and Integrated Biology Pavillon Charles‐Eugène‐Marchand 1030 Avenue de la Médecine, Université Laval Québec G1V 0A6 Canada; ^6^ Centre d’Étude de la Forêt 1030 Avenue de la Médecine, Université Laval Québec G1V 0A6 Canada

**Keywords:** *Ephedra sinica*, Ephedraceae, expressed sequence tag–simple sequence repeat (EST‐SSR) marker, genetic diversity, gymnosperm, medicinal plant

## Abstract

**Premise of the Study:**

*Ephedra sinica* (Ephedraceae) is a gymnosperm shrub with a wide distribution across Central and Eastern Asia. It is widely cultivated as a medicinal plant, but its wild populations are monitored to determine whether protection is needed.

**Methods and Results:**

Thirty‐six microsatellite markers, including 11 polymorphic markers, were developed from *E. distachya* RNA‐Seq data deposited in the National Center for Biotechology Information dbEST database. Among 100 genotyped *E. sinica* individuals originating from five different population groups, the allele number ranged from three to 22 per locus. Levels of observed and expected heterozygosity ranged from 0 to 0.866 (average 0.176) and 0 to 0.876 (average 0.491), respectively. Allelic polymorphism information content ranged from 0.000 to 0.847 (average 0.333). Cross‐species amplifications were successfully conducted with two related *Ephedra* species for all 11 di‐ or trinucleotide simple sequence repeats.

**Conclusions:**

This study provides the first set of microsatellite markers for genetic monitoring and surveying of this medicinal plant.

*Ephedra sinica* Stapf (also known as Chinese ephedra or ma huang; Ephedraceae), a gymnosperm shrub, is distributed across southern Siberia, Mongolia, and China, and is found in arid areas and highlands, occurring on slopes, dry river beds, sandy places, or fields in mountainous areas (Lin et al., 2002). The species is reported as dominant in some areas, but little is known about its entire population size. *Ephedra sinica* has been used in Chinese herbal medicine for thousands of years (Fabricant and Farnsworth, 2001). The stems of most members in the genus *Ephedra* L. contain the alkaloid ephedrine, which is used for treatment of asthma and other respiratory ailments (Liu, 1989; Nam et al., 2003). Recently, *E. sinica* has become extensively exploited in a large market developed for nutritional supplements and stimulants involving this plant. *Ephedra sinica* is recorded on the International Union for Conservation of Nature (IUCN) Red List of Threatened Species (Bell and Bachman, 2011). The IUCN lists the species as Least Concern; however, wild populations still need to be monitored to determine whether protection is required, as a species of Least Concern may still be critically endangered within a particular region where numbers are very small or declining.

Recently, 29 polymorphic microsatellite loci were developed for a distantly related species, *E. gerardiana* Wall. ex C. A. Mey., by mining the whole‐genome‐skimming data from Illumina MiSeq sequencing (De et al., 2017). However, no DNA markers have been developed for *E. sinica*, limiting our ability to monitor its population dynamics and employ conservation genetic measures. The present study developed a crucial set of di‐ or trinucleotide microsatellite markers by mining an *E. distachya* expressed sequence tag (EST)–derived database. The EST–simple sequence repeat (SSR) markers developed here will enrich the genetic marker set for *Ephedra* species.

## METHODS AND RESULTS

A total of 4981 ESTs generated from mRNA sequencing of *E. distachya* were retrieved from the National Center for Biotechnology Information (NCBI) Expressed Sequence Tags database (dbEST) (accessed by searching with “(Ephedra) AND “Ephedra distachya”[porgn:__txid3389]”). Microsatellites with a minimum repeat number of five were detected for 324 ESTs with a minimum length of 200 bp. We obtained 203 unique EST‐SSR loci by an all‐against‐all BLAST analysis and successfully designed primers for 171 unique EST‐SSR loci. All bioinformatic operations were performed using the microsatellite detection and development pipeline QDD version 3.1 (Meglécz et al., 2014). Finally, we selected 88 di‐ or trinucleotide loci with at least five repeats for further evaluation.

We sampled five populations (100 individuals total) of *E. sinica* in Datong, Shanxi Province, China (Appendix 1). Voucher specimens were deposited in the Herbarium of Beijing Forestry University (BJFC). In order to test for successful amplification of the 88 EST‐SSR loci selected, we conducted PCR analysis using eight individual plants of *E. sinica*. These eight individuals were collected in the Beijing Botanical Garden, Chinese Academy of Sciences. The genomic DNA was extracted from dried leaves using the cetyltrimethylammonium bromide (CTAB) protocol (Doyle and Doyle, 1987). An M13 tail (FAM, HEX, TAMRA, ROX) was attached to the forward primer (Meglécz et al., 2014) for visualization. The final PCR volume was 20 μL, containing 10 μL of 2× *Taq* PCR Mix (Tiangen, Beijing, China), 4 μL of fluorescent dye–labeled M13 primer (4 pM), 4 μL of mixed forward and reverse primers, and 2 μL (20 ng) of DNA. The following PCR conditions were used: 94°C incubation for 5 min; 25 cycles at 94°C for 40 s, 55°C for 40 s, and 72°C for 45 s; 10 cycles at 94°C for 40 s, 53°C for 40 s, and 72°C for 45 s; and a final extension at 72°C for 10 min.

Among the 88 identified di‐ or trinucleotide loci, 38 displayed the expected size bands. After final capillary electrophoresis analysis on an ABI 3730 sequencer (Applied Biosystems, Waltham, Massachusetts, USA), SSR alleles were called with GeneMarker version 2.20 (SoftGenetics, State College, Pennsylvania, USA). Of these 38 loci, 36 showed clear, single peaks for each allele as essential for confident scoring, and 11 of these loci were polymorphic among the initially screened eight individuals. Characteristics of the 25 pairs of monomorphic microsatellite loci developed for *E. sinica* are shown in Appendix 2. The 11 polymorphic primer pairs were subsequently used to screen five *E. sinica* populations (with sample sizes *n* = 20 per population) and two additional populations originating from *E. likiangensis* Florin (*n* = 20) and *E. equisetina* Bunge (*n* = 6) (Appendix 1). Table 1 shows the primer sequences, repeat motifs, amplification sizes, GenBank accession number of the target sequences, and functional annotations determined with the protein family database, Pfam (Finn et al., 2014). We employed GenAlEx version 6.5 (Peakall and Smouse, 2012) to calculate genetic diversity parameters. The allelic polymorphism information content (PIC) was calculated using CERVUS 3.0 (Kalinowski et al., 2007). Allele numbers ranged from three to 22, with an average of 11.55 alleles per locus. Levels of observed and expected heterozygosity ranged from 0 to 0.842 (average 0.176) and 0 to 0.883 (average 0.491), respectively. In addition, PIC values ranged from 0 to 0.847 (average 0.333). The genetic parameters calculated for the 11 polymorphic EST‐SSR loci are detailed in Table 2. The target sequences for all microsatellite loci are provided in Appendices S1 and S2.

**Table 1:** Characteristics of 11 polymorphic microsatellite loci developed for *Ephedra sinica*

Locus	Primer sequences (5′–3′)	Repeat motif	Allele size range (bp)	Fluorescent dye	Function annotation^a^	GenBank accession no.
E‐2	F: GAGAGAAGGCAAGTGTCATGG	(AGG)_6_	192–231	FAM	Peroxidase	JG722437
	R: CCATCCTCGTCTCTTTCTGC					
E‐18	F: AGTCGAAGCAGAAGGCTGAC	(AAT)_6_	153–228	TAMRA	Dev_Cell_Death	JG719586
	R: TCCTGGGAAGAGACTCCGTA					
E‐20	F: GATTAGGTGGAAAGCAAGCG	(AAG)_5_	164–170	HEX	DUF260, Oxidored_q1	JG721857
	R: ATCCAACCCGATCATGTACC					
E‐33	F: TTGATGATGTCTGTAGCGGC	(ATC)_6_	186–246	ROX	MGS, AICARFT_IMPCHas	JG720119
	R: AGTGGCAGAAGTGTTGGCTT					
E‐35	F: GGTGTTTCAGATGCGATTCA	(AAG)_6_	182–188	FAM	CK_II_beta	JG720356
	R: ATCGTTGATCCTCTTGCGAT					
E‐49	F: CCTTGAGGCGCTTTATTCAG	(AGG)_5_	175–253	TAMRA	MIT	JG721444
	R: CGCAAGATCGAAATACCCAT					
E‐58	F: GCTCTGTCGAGAAGAACCGA	(ATC)_5_	149–200	HEX	U‐box, zf‐RING_LisH,DOPA_dioxygen	JG722187
	R: GGGTGGAACTTGAGGTCCTT					
E‐59	F: GGATCCAAGATCTGGAAGGAG	(AGG)_9_	174–246	ROX	YycI	JG722338
	R: AAGCCCATGTCATCATCCAT					
E‐62	F: TGAATAGAAGCTGGCTGGGT	(AAG)_5_	173–248	FAM	No hit	JG722724
	R: TTGGCTGGTTCTGTCTGATG					
E‐71	F: AAAGCGTGCAAGACGAGTTT	(CAA)_3_CGAC(AAC)_5_A	171–261	ROX	AAA_assoc	JG723111
	R: TCCTCTTCCTCTCCACCTCA					
E‐83	F: GTCATGTCATGCTCACCGAC	(ATC)_5_(TTC)_3_	255–264	HEX	Pkinase, Pkinase_Tyr, Kdo, APH, RIO1, YrbL‐PhoP_reg	JG719186
	R: GCGACTTCTCATTGCTCTCC					

a Pfam annotation refers to the protein functional annotation.

**Table 2:** Values for genetic diversity of *Ephedra sinica* across 11 polymorphic microsatellite loci.^a^

Locus	MH‐1 (*n* = 20)				MH‐2 (*n* = 20)				MH‐3 (*n* = 20)				MH‐4 (*n* = 20)				MH‐5 (*n* = 20)				Total
	*A*	*H*_o_	*H*_e_	PIC	*A*	*H*_o_	*H*_e_	PIC	*A*	*H*_o_	*H*_e_	PIC	*A*	*H*_o_	*H*_e_	PIC	*A*	*H*_o_	*H*_e_	PIC	*A*
E‐2	2	0.050	0.050	0.048	3	0.000	0.272	0.247	2	0.000	0.185	0.164	2	0.000	0.097	0.090	3	0.100	0.188	0.174	5
E‐18	6	0.000	0.813	0.757	6	0.083	0.750	0.686	4	0.000	0.598	0.531	5	0.176	0.727	0.657	11	0.250	0.883	0.847	18
E‐20	3	0.167	0.379	0.337	2	0.111	0.489	0.362	2	0.000	0.097	0.090	2	0.000	0.097	0.090	3	0.100	0.272	0.247	4
E‐33	10	0.278	0.521	0.495	9	0.278	0.608	0.564	7	0.150	0.500	0.465	7	0.250	0.786	0.739	8	0.471	0.832	0.783	20
E‐35	3	0.105	0.104	0.099	2	0.200	0.185	0.164	2	0.050	0.050	0.048	2	0.200	0.185	0.164	2	0.100	0.097	0.090	3
E‐49	5	0.263	0.290	0.271	4	0.842	0.597	0.502	4	0.800	0.581	0.512	4	0.600	0.483	0.433	5	0.750	0.564	0.503	8
E‐58	4	0.105	0.711	0.636	4	0.125	0.762	0.689	3	0.067	0.398	0.351	7	0.000	0.823	0.770	5	0.000	0.694	0.627	10
E‐59	6	0.471	0.770	0.707	4	0.050	0.594	0.497	8	0.235	0.820	0.772	4	0.105	0.545	0.454	5	0.200	0.645	0.558	16
E‐62	8	0.000	0.876	0.834	8	0.211	0.751	0.695	10	0.235	0.845	0.804	10	0.350	0.836	0.794	10	0.300	0.831	0.787	21
E‐71	9	0.278	0.708	0.669	9	0.263	0.875	0.835	8	0.250	0.866	0.810	7	0.125	0.730	0.672	6	0.300	0.590	0.547	22
E‐83	2	0.000	0.097	0.090	1	0.000	0.000	0.000	3	0.000	0.190	0.177	1	0.000	0.000	0.000	3	0.053	0.152	0.142	4

Note

*A* = number of alleles; *H*_e_ = expected heterozygosity; *H*_o_ = observed heterozygosity; *n* = number of individuals; PIC = polymorphism information content.

a Voucher and locality information are provided in Appendix 1.

Furthermore, we conducted cross‐species amplification of the 11 polymorphic primer pairs on two related species: *E. likiangensis* from Yulong, Yunnan Province, and *E. equisetina* from Datong, Shanxi Province, China (Appendix 1). All 11 primer pairs successfully amplified *E. likiangensis*, except for locus E‐20, which produced monomorphic bands in the species (Table 3). For *E. equisetina*, nine out of the 11 primers tested were polymorphic, and two loci failed to amplify. The interspecific amplification profile may be partially related to the phylogenetic relationships between species, as the relationship between *E. equisetina* and *E. sinica* is more distant (Ickert‐Bond and Wojciechowski, 2004). In terms of polymorphisms, except for primers at the E‐49 locus, the remaining primer pairs showed moderate polymorphism in *E. equisetina*, possibly due to the small sample size.

**Table 3:** Cross‐amplification of 11 polymorphic microsatellite loci developed for *Ephedra sinica* in *E. likiangensis* and *E. equisetina*.^a^

Locus	*Ephedra likiangensis* (*n* = 20)						*Ephedra equisetina* (*n* = 6)					
	*A*	*N*	Allele size (bp)	*H*_o_	*H*_e_	PIC	*A*	*N*	Allele size (bp)	*H*_o_	*H*_e_	PIC
E‐2	4	19	189–237	0.526	0.528	0.444	1	1	182	0.000	0.000	0.000
E‐18	4	20	195–246	0.500	0.581	0.511	1	6	195	0.000	0.000	0.000
E‐20	1	20	185	0.000	0.000	0.000	2	6	170–185	0.833	0.530	0.368
E‐33	9	20	162–246	0.150	0.826	0.781	1	5	147	0.000	0.000	0.000
E‐35	3	20	182–277	0.400	0.337	0.289	1	6	188	0.000	0.000	0.000
E‐49	6	20	169–229	1.000	0.686	0.626	3	6	175–184	0.667	0.530	0.424
E‐58	5	19	152–197	0.526	0.627	0.546	1	6	195	0.000	0.000	0.000
E‐59	4	19	185–212	0.316	0.587	0.479	2	2	202–208	0.000	0.667	0.375
E‐62	5	20	150–224	0.200	0.486	0.438	1	6	222	0.000	0.000	0.000
E‐71	5	20	125–151	0.450	0.619	0.559	1	4	226	0.000	0.000	0.000
E‐83	3	19	247–265	0.368	0.317	0.275	2	6	189–258	0.333	0.485	0.346

Note

*A* = number of alleles; *H*_e_ = expected heterozygosity; *H*_o_ = observed heterozygosity; *n* = number of individuals sampled; *N* = number of successfully amplified individuals; PIC = polymorphism information content.

a Voucher and locality information are provided in Appendix 1.

## CONCLUSIONS

The EST‐SSR polymorphic markers developed in this study will be potentially useful for studies of population structure and genetic diversity in *E. sinica* conservation genetics. These new markers will also be applicable for *E. likiangensis* and *E. equisetina* and can enrich the number of DNA markers available for *Ephedra*.

## DATA ACCESSIBILITY

Expressed sequence tags used for primer development were downloaded from the National Center for Biotechnology Information (NCBI) Expressed Sequence Tags database (dbEST). GenBank accession numbers for target sequences of both polymorphic and monomorphic SSR loci are provided in Table 1 and Appendix 2.

## Supporting information


**APPENDIX S1.** Monomorphic microsatellite target sequences from microsatellite marker development in *Ephedra sinica*.

**APPENDIX S2.** Polymorphic microsatellite target sequences from microsatellite marker development in *Ephedra sinica*.

## Appendix 1 Voucher and locality information for *Ephedra* species used in this study.^a^

**Table:**

Species	Population code	Voucher specimen accession no.	Collection locality	Geographic coordinates	*n*
*Ephedra likiangensis* Florin	—	ELF201807281^b^	Baishui River, Jade Dragon Snow Mountain, Lijiang County, Yunnan Province	27.13205°N, 100.248755°E	20
*Ephedra equisetina* Bunge	—	EEB201807301^c^	Datong, Shanxi Province	39.95878°N, 113.776324°E	6
*Ephedra sinica* Stapf	MH‐1	ESS201806101^c^	Fan Yao village, Yanggao County, Datong, Shanxi Province	40.28975°N, 113.648139°E	20
*Ephedra sinica*	MH‐2	ESS201806232^c^	Nan Tuo village, Duzhuang township, Yunzhou District, Datong, Shanxi Province	39.95890°N, 113.776347°E	20
*Ephedra sinica*	MH‐3	ESS201806233^c^	Yang Lao Wa village, Xubao township, Yunzhou District, Datong, Shanxi Province	40.85174°N, 113.852189°E	20
*Ephedra sinica*	MH‐4	ESS201806234^c^	Longhun Mountain, Kang Yao village, Yanggao County, Datong, Shanxi Province	40.26208°N, 113.622244°E	20
*Ephedra sinica*	MH‐5	ESS201806255^c^	Bai Deng Mountain, Pingcheng District, Datong, Shanxi Province	40.12804°N, 113.372931°E	20

Note

*n* = number of individuals sampled.

a All voucher specimens are deposited in the Herbarium of Beijing Forestry University (BJFC), Beijing, China.

b Collector Yong‐peng Ma.

c Collector Dong‐xu Zhang.

## Appendix 2 Characteristics of 25 pairs of monomorphic microsatellite loci developed for *Ephedra sinica*.

**Table:**

Locus	Primer sequences (5′–3′)	Repeat motif	Allele size (bp)	Fluorescent dye	GenBank accession no.
E‐1	F: CCGAATCAATCGCTCTCTTT	(CT)_5_	151	FAM	JG721273
	R: GCCTGGTTCTCTCCCATTT				
E‐6	F: CAGTCAGGTCTCTTCGCCTC	(CA)_9_	200	TAMRA	JG723006
	R: TGCAACCGTGATATGAGAGC				
E‐12	F: TAGCTTGTGGCTATTGCCCT	(TAG)_5_	144	HEX	JG719000
	R: ACCCTCCTCCTCCATTGTG				
E‐13	F: AATCAACTTGGCCCAGACAA	(CAT)_5_	151	ROX	JG719115
	R: CCTCTTGCTTAGCAGCGTCT				
E‐19	F: GAAGCAGGAGCAGAAGATGC	(GCA)_5_	194	FAM	JG720107
	R: TTTGGAGGTCGCTGATGG				
E‐21	F: TTTGTGGTGTTGCTGACAGG	(AG)_24_	244	TAMRA	JG719754
	R: ACTCCTCTGCCTCCACTTCC				
E‐22	F: AGGCTGTGCAGGAACATCTC	(GGC)_6_	230	HEX	JG723316
	R: GTGAGCGGGAATGAGTAGGA				
E‐23	F: TAAACGACGGGTTCTCTCCA	(TG)_5_	173	ROX	JG719685
	R: TCAAAGTCGTCGAGGAGGAG				
E‐25	F: GAAACAGGCACAGACACGAC	(GGCACA)_5_	186	FAM	JG719706
	R: GATTTCCAGATCCATTATGCG				
E‐26	F: TGTTCCTCTCTCTGCGGATT	(TTC)_5_	115	TAMRA	JG719755
	R: TCCTTTGGAAGCTGACCAGT				
E‐30	F: ACACCACAGGCGAAGAAACT	(CTC)_5_	186	HEX	JG720051
	R: GGAACGGACAGTTGGAGAAG				
E‐36	F: ATTGAGCACGCAGACACAGT	(TTG)_5_	243	ROX	JG720438
	R: GTTCTCGGACAAACTCAATGG				
E‐38	F: TGGTCTTGGTCTCATCCCTC	(AG)_3_(CAC)_5_	228	FAM	JG720528
	R: TCTCACCAAATTCCCACACTC				
E‐39	F: AAGCGAATGGCGTATAATCG	(AGG)_7_GCA(AGG)_3_	249	TAMRA	JG720562
	R: AGAGGAAGCAACCAACCCTT				
E‐41	F: TAGAAGGAGGCGAGAAGCAG	(AGA)_5_	214	HEX	JG720763
	R: TAGCTGAGTCGATCCCACG				
E‐46	F: GGCAAACAGAAGGAACGAGA	(ATG)_5_	144	ROX	JG721163
	R: TTGCTTGGGTAATAGGCATTG				
E‐47	F: AACTGGACATGGAGGAGGTG	(TCA)_5_	222	FAM	JG721187
	R: AGAGCGTCAGCCTCAGAAAC				
E‐54	F: TTCCTGCTTCTTCTAATGCTTTG	(TGC)_5_	165	TAMRA	JG721879
	R: TCGGATCAACACCAAACTCA				
E‐55	F: AGGCCTTTCTCCGTGTGC	(GCA)_6_	253	HEX	JG721940
	R: GAGCAATGGCCTTGACGTAG				
E‐60	F: CTTGCAAGTTGCCGAAGC	(GA)_3_T(TTG)_3_(TTA)_6_	167	ROX	JG722642
	R: GGTGAATCCATCAAACGCAT				
E‐61	F: GGATAGGACCCGGGTTAAGA	(TA)_10_	249	FAM	JG722646
	R: GCTGCCCATTAACAAACCAG				
E‐65	F: TGCATAGAACAGTTGCAGAGG	(AG)_5_	223	TAMRA	JG723017
	R: CAAGCATCTTTCCAACCCAT				
E‐74	F: CAAATCCCTTTCTTCTCAGATTG	(TAT)_5_	193	HEX	JG723206
	R: GGGTTTCTTACCAGTTGCAGA				
E‐84	F: TCACTCTCTACAATTCATTCACAGC	(TC)_5_(TA)_4_	183	ROX	JG719254
	R: GAAGCCGACGTGGATAAGAG				
E‐88	F: TGACCAAGCTCAAGCAAGAA	(ACA)_6_	166	TAMRA	JG719585
	R: GAAGCGATGATCAGTGGTGA				
