# Supplementary material for: Development of novel EST‐SSR markers for Ephedra sinica (Ephedraceae) by transcriptome database mining
Source: Appl Plant Sci. 2019 Jan 16;7(1):e01212. doi: 10.1002/aps3.1212 (PMC6342172; doi:10.1002/aps3.1212)
Supplement: Supplementary file 1 — APPENDIX S1. Monomorphic microsatellite target sequences from microsatellite marker development in Ephedra sinica. [file APS3-7-e01212-s001.docx]

APPENDIX S1. Monomorphic microsatellite target sequences from microsatellite marker development in *Ephedra sinica.*

>E-1

TTTTAACCGAATCAATCGctctctTTCNTCTTNGTTTTCTTCTNTCTTTGCctctctctctNNTGCAGAGCTA

NCCTTGCAATATCAACTTTCCTtatataagagagagGGTCTCNAAGAGAATCGAAGCGATGTCGAAAATGGga

gagaACCAGGCCANTCTTCTACTCAGAAAACAACTCAAAGAGTTGACGAAGAATCCTTTGGATGGATTCTCAG

CCGGGCTTGTTGATGATTCAAATGtttttGAGTGGAATGTTACCATCATTGGACCTCCAGATACNTTATATGA

AGGTGGAttttttAATGCAATCATGAGTTTTCCACCAAACTATCCAAATAGCCCACCAACGGTGCGGTTCACA

TCAGAGATGTGGCATCCAAATGTTTANCCTGATGGGCGTGTTTGTATATCAATTCTTCATGCTCCTGGAGATG

ATCCAAATGGCTATGAATTGGCTAGNGAACGTTGGTCACCTGTCCATACGGTAGAAACAATTCTNTTAAGTAT

CATATCAATGTTGTCAAGCCCAAATGACGAATCACCTGCAAACATTGATGCAGCGAAAGAATGGCGGGAGAGT

AAAGAGGATTTCaagaagaagGTTAGCCGCATTGTCAGGAGGTCCCAAGAGATGCTATgagagaTTGAAATNG

CATGNGCNGTTTTGTATTNCNGGATCATTTTGTCCTGNTTTGNAAANaaaaaaaaaTGTTTACCGCNACTATA

TCTGTTTTGATGGGTGAAGTTTAATTTGATAATCTTTTCAGTATTTGTGttttttNCATGGACATTTGCTTTG

AAAATGCTCCTTAAGAAGCATTTGTGGATGTTAATGGCAAAATCAGCATATCACA

>E-6

AACTATAGCACAGTCAGGTCTCTTCGCCTCTCCAACACTAAAATCCCACCTTTAGGGTTTTAGTGCCAACTCT

GCATTTTCCTCTtatataCctctctTCCCcacacacacacacacacaCTCCATCAATTCCTTCTCTTACTTGC

CGTGCCCTTCCGTGCCGAAGCGATCGTTCTTCTCATAAAGTAGAGCTCTCATATCACGGTTGCAATGGTTAAG

TCTGCAAAGAAGGGTGCCACAGAGGTTGCTCTCCCTCAGTCTGGAAAGAAAGGAGGGAAGAGGCAACTGGAGG

CTGCCTTAGGGGTTAGTCCAGCTTCCaaaaaaTTGAAAACCGAGaaaaaaGCACCGCCTCCAAGAAAAGTGGA

GGCATCTTCAGATTCTGAATCTGATGATTCATCTTCAGATGAAGAGGAAGAGATTACAGTGaaaaaGCCAGTC

CAGGCAAGAAATGGCAATGGCAAGCCAGCTCTTAATGGAAAGGCGAGCTCAAGCGATGAAACTTCTGATGACA

GTTCTGACTCTGACAGTGATTCCAATaagaagaagCCCTCTGCTAAGACAGTTAAAGCTCCTGCCAAGAAAGC

TGATACTAGCTCTGATGAGTCTGATTCANAGGTGGATTCTGATGAAAGTGATGAAGAACCAGCTCAAAAGAAG

CTCAAGAATGAGGCAGCCAAGCCAGTACCTTCAAAGTCTACAAAAGACTCTAGCAGCAGTGAGGAAGACTCTA

GTGATAGtgatgatgaACCCAAGCCCAAGTCTAAACAACCTGCAAAGACCctctctaaaaaGTCTACTAAGGC

TACAGTGAAAGCTCT

>E-12

CAAGCTTCTACTGCATAATAGATAGCCTTGAAATCTTAAGGGAGCTTAGCAACAATGGGTGACAAGTCTAGTG

CCATGGCCATCTTGGTGTTGTTGGTGGCCTCCGTGATGCCTTCAAGCATAGCTTGTGGCTATTGCCCTccccc

TAgtggtggtggtgGCAACAATGGAgggggTGGTCATAAtggtggtggtggtggTAACAATGGTGGCCACAAT

GGAgggggCGGTGGTAACAATGGTGGCCACAATggaggaggaGGGTCAGGAGTGAGCCCCATAGTAAGGCCTC

CAGGAGGCTCTGGCTCTCCtggtggtggCTCAGGAGGCTCTgggggTAGCTCTGGCTCTCCtggtggtggtgg

CTCAGGAGGGTAtggtggtggTGTGCCTTCAAACCCTGGGTCAGGGTCAGGCTCAGGAGGCAAAgggggTGGT

GGTTCGTATGGTCAGAAGTATTGCCCAAAGGATGCACTCAAGTTGGGAGCCtgtgtgGATGTCCTTGGAAGCC

TTGTGCACCTAGTGATCGGTGACCAGGCGGTGAACAAATGTTGCCCTCTTGTCCAAGGGTTGCTTGAGCTTGA

GGCAGCTCTGTGCCTGTGCACTGCCCTCCGTGCCAAGGTGCTCAACCTTGACATCTATCTGCCCATTGCCCTT

GAGCTCCTTGTCACTTGTGGCAAGAACATACCGCCGGGGTTCAAGTGCCCTCCTTCCTCTGGCTACTGAAGAT

CATTCAAGATTTCGAAGGTTTTGGATTGAATTCAACATTGAAGAAAGCTAGCTAGGGATTGTGGCCATTCACG

AGAGTGCATCAtttttCATGTTTCGTCAGCAACTATTGtgtttgtttgtttgttGTGGAtttttATCCCATTG

TTATTAAATCGGACGACCATCTAAACGtttttAACTTT

E-13

TCAGACCTTTGAGCCCATTTAACTTCAGGGTGTCTACTCATGGTGTTGCAGAATCGGATTaaaaaaaGAGGGG

CTTTTGCAAGAACAAGATGCTAGATGNNNNTCcacacaCgggggATCTCACTCTCATTCTTCAAGATGGATag

agagAAGCTTATGAAAATGGCTGGTGCTGTTCGCACTGGTGGAAAGGGTACCATGCGCAGAAAGAAGAANGCT

GTTCACAAAACCACTACCACTGATGACAAAAGGTTGCAAAGCACCTTGAAAAGGATAGGAGTCAACTCAATAC

CAGCCATTGAAGAGGTTAATATTTTCAAAGATGACTCAGTGATTCATTTTGTAAATCCNAAAGTTCAAGCATC

AATTGCTGCCAACACCTGGGTGGTCAGTGGAGCTCCACAAACAAAGAAGCTTCAAGATCTNCTACCAGGGATT

ATCAATCAACTTGGCCCAGACAATTTTGCAAACTTGAGGAAACTTGCTCAGCAATTTCAGAAACANGAAGCTC

ATCCACCTNCTGAGgatgatgatgatgatGTGCCTGAACTTGTTGAAGGAGAAAATTTTGAAGACGCTGCTAA

GCAAGAGGCCACTGCATAGAGGAACTTTAAGATTTACGAGTAAATGCTNTTGTCTTGAAATGTGCTNATCCTA

TTTAGCTTCATTCAGGGAGAATTTATTCCTATTTTAAGTTATGATACAATGCTGTTTTCTCTACCATCTCACA

AGGAAATCTTAtttttttGGATGTATTGAATACGNTTTAGTCCCAACTTTTACTTTCTGTGAT

E-19

AGGAAGGTAATGATCTGAAGCAGGAGCAGAAGATGCAGCAGCCTCCTTCCATGATGAGAAAGCAGCCACCATT

CCGgcagcagcagcagcaACAACAAAGGCCGCCACAGCAGAGGAAGGCGCCGGACCCGGCGGAGCTGATAGCA

ATGTACGAGGCGAAGGGCATGAACCCTACCGAGGCGTCGCACAAGGCCATCAGCGACCTCCAAAAGGCGCTGT

CGtctctcATGGGTCGCCTCCAGACCGGCAAGAAGCAGCCGAACGACCCCGCGTACACTTCCGCCCGCGTCGA

GCACATAAACCGGCGCATCTCCGTGCTCGAACTGAAGATGGACACGAAGCCCAACATCCCTCAGACCCTCGCC

ATAGGTGTCACTGCCGGTGCCGTCGTCAATGCCGTcccccACATCTTCAAGGCTCTTGCCGGCATGCTGTCGT

CTTTCCGCAACAACACCCGCAACACCTGACCCGGTACCGTCTTCTACCCtttttGGTAtttttGGGTACCCTT

TTGCGAGATATTTTGGTGGTCTTGCTTAGAATTTACTGAATTCGGCCAGGTTTTCCAATATTATGATCATAGG

GTTTTGGGTTTTGTTGTTCATGGCTGAAAGATGATGTAGATTTAGGGTTTTGGGtttttATGTCTATGGGTGT

AAGATTAACTGGTTTTGGGGTTTAATTAAGGATGCAATTAGAATTTTATTACTCTGCTGTAATTGAGGGCTTA

ATGGTCtttttttCTGATTCCACTGTTGTGGAAGAtttttGTTGTTAAATACATAACATAAATTTCATTAGAA

AATTAttttttCAATATTGT

E-21

CAAATCGCTGTGGATAATAACAATAAAACTCACTCCGAATCTTCATCAATAAAATTaaaaaaaaTATAATTAA

ATCGCatatatatACAAGTATACAGAAATAGAAAGATCGAAGAGCAACAAGATCATAATTCATTTGCAGAATT

GCAGTCGCAGTTGTTGCAGTCAGGGAGCAGCAACAAGAAGAAAGAAACTATCAGAGTGCCATTTCTTACTGCA

GAAATTGACAGAGTGACAGATGCAGGAGAtttttGCGTGAGGTCTTAAATGACGCCTCAGAGCCTTTTGTCCG

ATGCGTAGATGTCAATCTGCAAAGAAAGGGAGAAATAAATCGCActctctctATTGGCTAtttttGTGGTGTT

GCTGACAGGGCTTATTTTGTAGTCTTTACGCTCAGATCTGAAAGAGAAGTATAAGAATGGAATTAGTCATGAA

GAATCCTAGGGTTTGTTCACTTTGAAGTCTGtttttATTTCCAGATTGAagagagagagagagagagagagag

agagagagagagagagagagagagGGTGTGGGTGTAAGTTTTGAGGTAAAAGGATGCAGACTGAAGAGGAAGT

GGAGGCAGAGGAGTACAGGCCATGGGACGAACTCATCCCTGATGCATTGTCAATTATTTTCaaaaaaaTGTCA

CTTCAGGAGGTTCTCACTGTCGTTCCTAGCGTTTGCAAGTCATGGCAAAAGGCTGCTGCCAGGCCTGATTGCT

GGCAAGTGATTGATATTGAGGATTGGAGCCGCCGTTGCAAGCTGGAGAACACGGACAGAATGGTTCGCTTGCT

CGTTGGCCGTAGCCGTGGTTGTATGTGGAAGCTTGCtgtgtgCGGCCTCAAGAATGACTGTAGCCTTGGTTTC

ATTGGCAAACATGGTCA

E-22

TCCAACAGTGCCGCGAATTCCCGTCTACTGACGCCCAATTACTAacacacacCAATTTCACCTCGCTcacaca

GATAGATCTCTTATCATcaacaacaaAAATGAACCAAGTATCGCATACACTTACATTTCAGCTCTGCAGTTAG

CTCCgtgtgtGGCGATGTGAAGGCCGAGGAAGaaaaaCATGGAAGGGATgggggACAACAgggggAGCTTCAC

GGATCTACTTATGCAAGGCTGTGCAGGAACATCTCCCACTATAAAgacgacgacCCCTCAAGGTGCCGGGAAG

GCACCGAATTCCCTTCAGAACGGACTCAATTTCTCTAAACCGCCTCCTTTTCCTCCTTACGGAggcggcggcg

gcggcggcGGACACAGTCACGCAATGCAAAATCACAATCacaacaacaacaTGTTTATAAATGCCACCATTCC

CCATATTCCTACTCATTCCCGCTCACTTTCCCAGCCTTCCTTCCTACAAAACCCCGCCTTCACGGAGTCCCCA

TCTCCTTTCTCAGCTCCTGCCACTCTGAAGCCCCGTGAGGCTCCGAGACCGAGGGAAAGCAACAATCCGCCGA

GGCACCGGCGTTCGCATAGCGAGATCAATCTTCCTATCAAGAAGGAGTCCGGGGACTGGGACAAGGACTGCGA

TGGCGACCTCAATATGGACGCCgggggCAGCGGCGGAGCTGGTGATGACTTGATTTCTTTGTATATCGACATG

GATAAGATCAACAATCTCAATTCTGTTACTGAAGGGAGCGGCGAGAGCGGTCGCAGAGTTGTTGATGAGAatg

atgatgAGGATAGCGCTGGGAATAGTGTCAAGGAGAAGGACCTTGATAGTGGCGATGATGCTGAGAGCGAGTG

CAATGAGAAGAAAGAGCGCCTCAAACGGAGCTTCACTGGTGAAAGATCAGGTGGAGGATACTCCCATCATGTG

CGGAGCTTGTCAATGGACAGTGTGCTGAGCAATTTTAACTGTAGCAGTGGTGAGAATAACAAGTCTGCGGCAC

CTTCACCTCCTGGTCAAAATGCCAAGCTTTTCCACAGCAATTCCATGGACGGTAATGCTAATTTCAAGCTTGA

GTTTGGCAATGGTGAGTTTAGTGCTCCTGAGCTTAAT

E-23

AAATCCATGCTTAGCAAGAAAAGATTAAACGACGGGTtctctcCATTTCCATCCTGAAGAGATCATCTtgtgt

gtgtgTCAGTTTTATTACTGATCCAGGGCCATGAATAACAACGAAGAAGAGGAAGACGAGGGACTGCAACTGT

TCCACGATCTTCACAATGACCACCACGCCAGTctcctcctcGACGACTTTGAGCTCCCTCCAGGTCCAGGGGA

AGACAAACTTAGTAGCTCTCCAATTCTTCAGTTTTCAACACCCTCCTCTGGGCGGAAGGGAATGCTTGATGAT

TTTCTGAAAGATAGTGGTaaaaaTGATTATGACTGGCTTCTAACACCGCCTGGCACTCCATTATCTCTGTCAG

TGGATAATGAACCTACCAAGACATCTACTGAAAAGAAAAGCACGTGTACAAGGGTTCTCCCTGCATTAAAGAC

TTCAAGGCTTTCTAAACCTCAAACTGACTCGACATCAAGGAATATAGCAAACGGCTTCAGATCTCAGCCAGGA

AGTCCTAGCAAAAGTGGGCATGGTTTCACTTcaacaacaaACACTCAAGAAAACAAGGCAAGGAAATCTTCAA

CACCTAAATCTGCATCTTCTTCGAGATCTTCCACTCCTGTTTCAAGAGCAAATCTTTCATCACGAAGACCGTT

GTCAAAGCCACAAACCCCATCTTCAGCAACACATCTACCACGAAGGCCATCAACTGCTTCCTCAGCCTCTAGG

CCATCAACACCACCCTCAACATCAGTAACAAGTCAGaaaaaaCCTTCAAGTCGACCAAGATCCTCTTCCACTG

GCAAAACCACTGCTATTCCGTCAAGGCGGG

E-25

TAGGCACGCACGGAAACAGGCACAGACACGACCACGACCACGCGGCCGGGACTggcacaggcacaggcacagg

cacaggcacaGGCACGCACAAATAGTTATTGATTGAGTGTAGAGGAGGCAAAAGAGATGCAGTTGTTACCAGA

GAAGTGGTTTGAGAAGACCCagagagAAGGGCGCATAATGGATCTGGAAATCCTcccccGCgaccgagaccga

gaccgaGACCGCGATTCGGAGATGCAGGCGCCTCCGGACGAGTCACTGACGGTGCTTCAGCTgagagaaaaaa

TCGACAGCTTGCAAAAGTATCTTAACACCATGAGTTTCCCGGACCGTGGCCAAAAGCTTCGCAACCGTATTCA

TATCCTCCAAAGCCTCCTCCAATCTAAGTCTAAGACTGGTGAGCAAAGGATGTCAACTGACTTTaaaaaGTTC

TCTATGTTTGGTAATCCCGAACAAGCTTTGCAATCTAGGGATGTTGATATGTGCGGCGTAGATGAAGGGAAAC

CTGCCTTTGATGTTGAAATTGGACACTTGAAATCaaaaaaTCAGTCAGTGTACACTAGTCCAATAATTTCGTC

CAATCGTTCTAACAGTCTAACCAGTATAGAAAGCCCAGCAGAATCAAACATTCCTGAATGTTCAAGAACAAGT

AGTTCTGGATGTCATGATTTTATGTTCCGCAAAGAGAATATTACCGTGAGGTCTACCA

E-26

GTCCACTTTTCCACGACGTTCTTTCCATAAATTGTCGTTCAATACTTTGAGAATTTCTTGTTCctctctctGC

GGATTCTCTGCAATATACTTCAGCTTTttcttcttcttcttcAAGATtctctcAAAGCTCTGCAATTCCAGaa

aaaaTGAACTGGTCAGCTTCCAAAGGAGGAGCTaaaaaTGGTATATCCATTCATGGGTCCAGCAAATATTTAA

TTACTGAAGGAGGAGTAAGGCATAATaaaaaGCAAGTTTTCCAAAGAGATGCAGAGAATGAGAAGCAAAATTT

ACCAAGGGATGAAGAGGACCAGGGTGTACCAAAGAGGAGAAAGCCGAGTCATGACTACCAAAGAGATGAAGAG

AACCAGGGCGCAGCTATGCGGAGGAAGTTGAGGCATGAATATCAAGACCTTAGAAACTCGATTCCTGTTCATC

ATGAAGAATGGCTTAACACAAGTTCAGGAAAATTTGAGGAGATCATTaaaaaaGTTGAAAGCTTGCACTCTTC

TGTGGTAAAGCCGCGTGAGCAGTTATCAGATGGTAAAATAATTCTGCAAATTGCACGTTCCCTTCGTGATTCT

GCTACAAAAGCGAGGGCGGCCAATGCACTGTCTCCATCAGATTTCATAAATGCAGTTTTGGCAAGCCAATTTc

acacaCCTAATGCAGCAACAACCTTAAAAGGTGCTGCTATTAAAGGTATTTCTTGGCGCAAATTG

E-30

CGCCAgtgtgtACTTGTTTGTTCAAGTGACGCACGCCTCTGCAATCTCCGCCATTTCAATCTGTTTGCCACGC

TCTTCCTCCCCGCAGACATACAATGGCGTCGTTCGTAGCTCTGAAGCTGTCGCTGGGAAAGACGCAATGCACA

ATGGCAAGCGCCATGGGGAACAAGAACACCAAATGGAGAACGAGGACAAAGGGGAGGgagagaATGAGGGTCG

TGGCGAAGGTGAGGGAGATTTTCATGCCAGCGCTCAGTAGCACCATGACGGAGGGCAAGATCGTCTCATGGAT

CAAATCCGAAGGAGACGTCCTCTCCAAAGGCGAGAGcgtcgtcgtcgtCGAGTCCGACAAGGCCGATATGGAC

GTCGAGACCTTCTACGACGGCATCCTCGCCGCCATCGTTGTCCCCGAGGGTGAGTCCGcccccGTCGGAGCTT

CCATAGGTCTCCTCGCCGAGACCGAGGACGAGATCTCCGAGGCCAAGGCCAGAGCCCAATTACAATCCCAACC

TTCTTCGATAAAACTATCCTCTCAGTCCGCCACTGCCACAGTGGACActcctcctcctcCGCCACCTCCTGCC

GCATCGCCATCTCCGCCTCCGTCTGCTCCGTCAGGGAAACTGGTGGCCACACCACAGGCGAAGAAACTGGCCA

AGCAACACAAGGTGGATCTGGCTAACATTGTTGGAAGTGGCCCTTATGGCCGAATCACTCCCTCCGATGTTGA

ATCAGCCGCTGGAATTTCTCCTAAACCCTctcctcctcctcctcAAGATGGcccccTTAAACCTTCTACTTCT

CCAACTGTCCGTTCCCCATCTCCGAAAGCGTCTGCCCCGTCTCCATCTCCATCCGCAGCAGTTGT

E-36

CAGTTGCTGGCACCAGGAGAAGATGTAGTCTGGGTCGGAGCTCTAGGCGTTGAAGaaaaaCAAGGCTGGCTCT

GAGCGTGACGGAACTAAGTTCGTGCGAATCCTACTGAAATAAATGAATGTGGCTCGACAACAAGGATGTCAAG

AGATCGACGGCTCCGACCAGGAGCACAAGAAGACCTCAATGGTACACCATATGTCTAGGAATGGGATGATGAG

TCTCGAAAGGCTGCTGGACCATGCAGAAAAGGAGTGCTAATGCAGAATATGAAAATTAAAGGAGACTGACACT

ATTGAGCACGCAGACACAGTGTAGCAAAGGAAGGGCGTTTGGGCGAGCTAGATGGTGGAGAATGACAACTTAA

GTGCCTAGCTTACAACATTttgttgttgttgttgACCTCAGCACATTAATTATATTCCTTGGTTCCttttttC

ATGTTGTCaaaaaTGTAATTCTGTTTATTGGAGATAATTTATTTCAAAGATGTAATTGCTTCAATCTATTAAA

ATGCCATTGAGTTTGTCCGAGAACaaaaaaTAGAAGCCATATTTGCTATGAAGCAATGAGGAATTTTGTTaga

gagTTTTGCTATACATGGTCTCCATTTCATAAGACATATTTGCTATGCAGCAATGAGGAATTTTGTTATAGAT

TTGCTA

E-38

CATGGTAAGCTTGCAGGAGCAAGAGGTGGTGTTGATGCAGAGGagaagaagaagaagaAGTAGTATTGAAGAT

GGTGAATgtggtggtgAAGATGATGAGACCTGGTCTTGGTCTCATCCctctctAGGTCTAGGACTTCATGAAC

aaaaaGACGCTTCACTTAATAATATACTTAAACACCATCCAGATCTagagagcaccaccaccaccacCATGGT

GGAGGAGGGAGGAGATAAGTTGGACAAAGTGATGACTGATATCAATGACATCTGGGGTATGCCTGACTGCAGG

GCACCCCTTCTCaaaaaaGAGTGTGGGAATTTGGTGAGAAGGTTGAAATTGCTAGTGCCCATGTTTGAAGAAA

TTAAAGAGTCCTTCATCATTGATGAACATCATGATAGTAATAATGATTCTATTGATAAGGAGAAGAGGAAGGG

CATCCTTGTTTCTTTGGACCAGTCACTCAAATCCATGCACTCTGCTCTCATTTCTGCCAAGGAGCTTCTCAAG

TATGCTAATGAGGGTAGCAAGCTTTTCATGGTTCTagagagGGAGCATGTAACAAGTAAAATGCATGATACAA

CTTCCCAATTAGAGCAGGCCTTAAGTGACCTGCCTTACTTGGAGCTTGGTCTTTCTGATGAAGTTTGTGAGCA

GGTTGAGCTCGTACATGCACAGTTCAGAAGGGCCAAAGGGAGGGTTGATATGGTGGATACAGAACTTGAAAAT

GACCTAAACATCCTTCTGTCGCAGAAAAGTGATAAAGATGACAATGATATTTTACGAA

E-39

GTAAGTAGGGATTGTTGATACCTGATTCCACAAAGAAAAGATGTCGAGCGTAGAAAGCAAAGACGAGGACGTG

GAGGCCATGTGACTTTGGTCTTTGTATGCTGCATCGGACGCCAAAACTCCAATTCAAAATGATTTTCACTGAA

ATTGCGGCCAACTATCCTTGATCCGtccttccttcctTCCCTAACTCTGAAGATGAAGCACCGTCCAGTCCAT

TCATTTCGTTTTCTTCTCCAAACCGAAAGAAAGAATCAaacaacaacaacaacaacATGAATGTGCCTACTGT

CCTGTAATTTCTCATCAtttttATCTTATGGTCGcccccACATATTTGTTTCTTATCCACCATTATCTTTATC

TTTAAATCACTTTCGCAATCAATTCCATCTTTAATATTAtttttCTTTAAAGCCAAAGAAATTCTGTGTTCTA

ATTTCTTTCGTTCTTCCGAGGCGAGTGATCtttttttttttCATGTCTTGATGTACAATCTAAGTCTTAAGCG

AATGGCGTATAATCGAAGGGTTGgtgtgtGATGGGAATTGGTagagagAAATAAGGAGGgagagaTGAGAGTG

TTGCCTTGGAACCAaggaggaggaggaggaggaggGCAaggaggaggACAAGTACAAGATCTTGAGCTGGCTT

CCTTAaagaagaagAACAAGTCCAGGTTTGCCTCTTTTCTTGTTTGTGTCTGCTGCCACTCTCCTTCCCACAA

ACCACAAGGGTTGGTTGCTTCCTCTTCACCACCCCTCAAGGAGGACACCAAGTTGGAGGGTGGTGCTGCCTCT

TCTTTGCTCCGCCGCGCCCTATCTCCCTCTCCTCGTGATGATAATGATG

E-41

GttttttAGTATAGAAGGAGGCGAGAAGCAGAGTTTTGTAGGGTTTGCAGAACAAGGAGcacacaACGTCAAT

CTCTTGCGAGagaagaagaagaagaAGCGATGGGTTTCAAGGGCACCAAGGCGGagaagaagaCGGTGTACGA

CAAGAAGATATGCGAGCTGCTGGACGAGTACACGCAGGTGCTGGTCTGCCTCGCCGACAACGTGGGATCGACT

CAGCTACAGAACATCCGCCAGGGTCTCAGGCCCGACTCCGTGGTTCTCATGGGCAAGAACACTATGATGAAGC

GCTCCATCCGTACCTACTCCGAGCGCACCGGCAACACCGAATACCTCAACCTCATTCCCCTCCTCGTGGGAAA

TGTTGGTTTGATATTTACTAAGAGTGATCTTAAGGAAGTCCGTGAGGAAGTTGCTAAGTACAAGGTTGGAGCT

CCAGCTCGTGTTGGTTTGGTTGCACCAGTTGATGTGGTTGTTCCACCTGGAAACACTGGTCTGGATCCATCCC

AAACTTCTTTCTTCCAGGTTCTTAATATTCCAACCAAGATTAACAAGGGAACAGTTGAAATTATTACACCAGT

TGAGCTTATCAAGCAGGGTGAAAAGGTTGGCTCTTCAGAAGCTGCATTGCTTGCCAAGCTAGGAATTCGTCCC

TTCTCATATGGGCTAGTGGTGAAATCTGTCTATGACAATGGATCTGTGTTTGATCCTGAGGTTCTTGACCTCT

CAGAGGATGATTTGATTGTTAAACTTTCTGCTGGCATCTCCGCAGTTACAGCTCTATCTCTTGCAGTCAATTA

TCCTACACTAGCAGCTGCACCACATGTTTTCATCAACAACTACAAGAATGTCCTTGCCATTGCTCTA

E-46

ACTTCACCGCTTCATAGTTTGCCATTAGCAGTAGCATATAACCTTCAGAGTTCAGACTTCCAAAACAAAATta

tataCACCATCGCGAAGTAGAAtctctcCGATATTTTCACAGGTTGATAATTTGATAAGCTAGAAGAATGATT

TCTGCtgtgtgTTGGCTTCCCAAAGGGGTTACAAAAGAAATACCAGATTTTGTAGAGCCACCTTCCCAAGAAG

AAATTCAGGAATATCTCAACTCCAATCTAGCAACAGATTTAAGCAATGGCAATGAAGATTCAGATGAAGAAAT

CGAGGATGAAGGCATGCTGGATGATGAAGAGGATGAAACTATTGATCCAGTAGAAAAGGCGCTGGCAGTTGCA

AATGCACTTGGAAGCGAGGCGGCAAACAGAAGGAACGAGAAAATGGAAGATATTTCTGAAGCTCTTAAAGAAC

TTGACATGGAGAATTatgatgatgatgatgAAGGAGAAGGCGTTGATAtttttGGAAAGGGGACCATAAGCAA

TGCCTATTACCCAAGCAATGACATGGATCCTTATCTTAAAGGCCAAGAGGAAGAAGATGAAGATGAAATTGAA

GATATGAGAATCAAACCTTCTGATTTGGTCATCTTATGTGCAAGGAATGAAGATGATGTCAGCCATCTCGAGG

TCTGGatatatGAAGAAGAGACCGAGGACAGCGAATCAAACATGTATGTTCATCATGATATAATCCTGCCAGC

ATTcccccTTTCTTTG

E-47

CGCAAAaccaccaccCATTAAATAGACCCCGATGCTACCAACACGATCCGGTATATTCAAAGTTGCAGAATAG

AACTGGACATGGAGGAGGTGAAGAAGCGGTGGATAAGGTGCCTCTTGTGGCAGCAGATGTGCGGCGCCCTGCT

CTGGCTGTGCACTTCGGCAACCCTAGCATCACTCtcatcatcatcatcaTCGCCGtatataAAAGGAAgcacg

ggcacgggcacggCCACAGGCACAGGAAGAGGCACAAgaggaggagCGGCGTgggggTTTCTGAGGCTGACGC

TCTTCGGAGCCTGCGAGGCAATGGTGATGGCCGCACACTGGatcatcatcTCTCCCGAGGAGAAGCGCGGCGC

GTCTCCGTTTGAGCTCTTGATGTCTGCGTTCCGCACCGGGGAGTATAAACTGCGCCTTAGGGTTAAGACTACC

GGGGACTGGATCCTTTTCATCGCCGTCTGCACCGTGAGCGCGTTTCTTCTCACtttttCTCTCGTCTCTtctc

tcCATCTGTTGCTCCGCGGCGCGGTTCTCGGATTGTTCTACGCGCTTCATTATGTGTACAACGGGCGATGGAT

TCTCCGCTTCCCGGTCATCCAGAGGCCTCCAttttttGGTTTTAAAATCGGTATTCCTTCAGCAGCCaaaaaa

GCTTTGATGTtctctctcATAATCACACCCGTCTCTGAAGCTGTGGTGCTTTTCCTTCTGGGCAACAGATATG

GTGAAGGaaaaaTGAAGtttttGAGTAATCAAATACTTTTCTTTCTTGAGTCATTTCTTGTATCATTTTGCTG

GGAGCTGACGCACCATATTATTCAGGTTGTGCATA

E-54

TTTTGCGAAAGGAAAACctctctCGACGATTTCTACAAGAGATCTAAACTCCATAGCCACTTCTTCATCACCA

TCTTCTGTACTTATCTTCTGAATCAGCAACCATGGTTCTTGAGGCGACTATGATTTGTGTAGACAATTCAGAG

TGGATGAGAAATGGGGATTATTCTCCAACAAGATTTCAGGCCCAATCTGACGCTGTTAACCTCATCTGCGGCG

CTAAAACTCAGGGCAATCCGGAAAATACAGTGGGAGCTCTCACCATGGCAGGAAAGAATATTCGTGTTCTGGT

CACCCCGACTACCGATCTTGGAAAGATTCTTGCTTGTATGCACGGGCTAGAGATTGGCGGAGAGTTGAATCTT

ACATCTGGTATACAAGTGGCTCAGTTAGCTTTGAAGCATCGACAGAATAAGAAACAACAGCAACGGATTATAG

TGTTTGTTGGCAGTCCAATTCAGTGTGAGaaaaaaaCTTTAGAAACAATTGGAAAGAAACTAAAGAAGAATAA

TGTAGCACTAGATATCGTAAATTTTGGAGAAGAGGATGATGCCAAAACTGaaaaaTTAGAGGCTCTTTTGGCA

GCAGTAAACAGTAATGATAATAGTCACATTGTACATGTTCCTGCTTCTTCTAATGCTTTGTCAGATGTTCTTA

TCAGCTCACCTATATTTACTGGAGATGGAGAAGGAAGTGGATTTGCAGCCGCAGCAGCTGCAGGtgctgctgc

tgctgcAGCTGCGGCAGCTGGTGGTGCggggggATTTGAGTTTGGTGTTGATCCGAATCTGGATCCGGAGCTG

GCATT

E-55

GAGCTTCATTAGCGTTCTCCTTGCCCGCGGTCCCCGCGGATGGCAGAACCGTCACGCTGGACCCTCCACGGTG

TCATCCTAGTCATTACCACGTAGGGCCGccccccAGGCCTTTCTCCGTGTGCACCCCACTCTCCAATTCTCTG

TCGCTGTCCACATGCAGAACCGCAGTTGCCTACGCAGCAACCAGCCAGAGCGAGTCCGACACGGTggagtcgg

agtcggagtcGTCCAGCACTgcagcagcagcagcagcaGAGAGTGAGGAGGACGATGTGTACGAGGTGGAGCT

GGCCAAACCCATAGGCCTTCAATTCTACAAAGGCTCAGACGGCGGCGTCTACGTCAAGGCCATTGCTCCCggg

ggTAATGCCGATAAGACCGGCAAGTTCACCGTCGGTGACAAGCTCCTTGCTACCAGTGCTGTATTTGGAAATG

AGATTTGGCCTgcagcagcaTATGGCCAAACCATGTATGCCATACGCCAACGAATTGGGACACtatataTGAA

AGTGCAGAAACGATATGGAAAGGTAGAAGATAACACAATGGATGAGAAAGCCATTATAGCAGCagagagGAAT

GCTGGGGTAATCAGTGATAGAGTGAGGCAACTTCAAATGCAAAATTCAATGCGaaaaaTGGAG

E-60

CCGACTCGCTCTTTGCGAATCCGCTTTCCCTCAAAACCCCTCTGATATACATCCATATCGTATCACATTCCGT

TTGAATCAAGTATTATTCTACTTGTCAGATTCCTCAAGATCACCCATAACCAAATATTCTTCTTATTCTTCTC

CTGAGACCTTTTCCTTTTCATCGATTCTATTCTCAGAAATTAGGTCATACAAACCAACAAAGCGAAATGTATC

TATaaaaaaaTATACACAGACACCAGTGACATCATACTCGGAGCTTTTaagaaagaaagaAAAGTGGAGTGAA

TTGCAAACTACGCTCTGTTTCTAAAATGGCATTAAAAGAAGGCCGCGgttgttgttgttAATCCATAAATACT

ttattattattattaTATGTTAATAATTACGTACTATGATATTTGTACCGCTACAATGGCCTGCCTGCAAATA

TTCCCTTGATTGTTGGTGGTGCTTATCAACAAtcattcattcatGATAATTCTCATTTAGCGTCGGTTCTCAT

CCTTCCAGCTTTGTACTTCctctctTTCTTTCGTTTCAGTCGGTGgtgtgtTAGTCTGTTTGTTTCTTATCct

ctctGtttttttCTTGCAAGTTGCCGAAGCtattattatAGTTCTTATTAGTGTTTGTACAGACCTTTGCGga

gagaTttgttgttgttattattattattattaCTTGTTACGGGAGGatatatTGTTTTGTACATTTGTGATtg

tgtgGTTGGAATAATGCGTTTGATGGATTCACCAGCTTGTGTTGTTGgagagaATAGTCAGGCAATGCAGATC

A

E-61

GCGAGGTCTGCATTCTAGGAGCACCGCAGAGGTCCTCAATGGCATTCACCCAGGTTTCACTCCTACAACTGTC

ATTACAAGCGGGGTATTGGACTTGCAGATGAGATACCTGCTGATCATGGGGTTTCAACTCAACTTCGCTAAGT

GGAAGAAAAGTGGATAGGACCCGGGTTAAGATGGGACTGACGCGTCCTCTAGAACTCTTTGTGGACAGGCATA

TGGAGTGCAACAATATCGTTCTCATGATGtatatatatatatatatataTGtttttGTAGTATTTGCAAtttt

tATTTCCTACTCATTCCTACTATAGAACAATAGTGCCTGTAAATTTTCCTTCtttttGTTTTGATTCATTTAC

TCACTAACTGGCTATTGGAAACTGGTTTGTTAATGGGCAGCAATGGCAAGCAATTTTCACTCTATTTTCATAG

TAGATTTGTGTCATATACATTGCAAGAGGTTTGGAGCCTTATCCTTCTATAGCCGAGGGGAGGACCTAAGGCA

ACAAGTAGTTTGGGGTAGATACTGACAACTAAGGGCTGCCCAAACATATAAGCAGTTACTTACTACTGGGATA

GTCGATGAACAGGGTTGTATTTGATGCATTGTATTCATCTATGACTATTCATAGTCTAGATGTGTAATAAGAA

AGCATCAttttttAATGACTGCAATCACATTCTAGTTTGTAATAGCATATTATTCATAAGTGTAATAGCATAA

AACTCTCAATTTGG

E-65

GATCCGAAATGGCGGAAGCTTGGAGCATGAGAGGTGTCAAGGCGGTGCTCGTTCTGCTCGCTCTCGGCCTGTC

CGTCTACATAGTCGGACCTCCACTGTTCTGGCAGTTCACTGAAGGACTCAACTTTATTAGCGCAACTTCCTCT

CCGTGCTCTCCATGTGACTGTGACTGCCCCTCCAGttcttcttcTTTGGGTTTAGCCAATCTTACTCTCCCAG

ATTGCGGGAACAATGATCCCCAGGTAAAGGTAGAACTGTCaaaaaaTTTGACAGACCTACTCACAGAGGAATT

GAGGTTGCATAGAACAGTTGCAGAGGAAAATCAGCAGCATTCTGAAATTGCTGTTCTTGAAGCTAAGAGATTG

GGATCTCAATATCAAAAGGAAGCAGAAAAGTGCAACGCTGGAATGGAAACATGTGAAGGGGCTagagagagag

CAGCAAAATTATTAGTCGGTGAGAAGAAACTAGCAGCCTTATGGGAGCAAAGAGCTCGCCAAATGGGTTGGAA

AGATGCTTGATTACTCAATGATCGCAAGTATGAAATGTTAGCCTAAATTATGGATTAGAGTTGGAGTGTAAGA

ACAGTAATTTATTTGTTGTATTAGatatatAGTTTTCATCCATCTCTAAGCTGTATGGAGACCTTTGAGATGA

TGAAATGAGGTTACCGATCGCTTGGCATGACTGTTATTGCATTGAAAACCCATTGAATTGTGCATAAAGTTGC

ACCTTGTACCATCTATGAAGTAAGCTATTATTTTCCTATATCA

E-74

CGGATTCTCAGACATGAAGGTAAGGGGAGAAGACGCATCGCagagagagACGGATAGCGGCTTGAGGTAGAGG

ACGAGGACCTATTGGCCGCCCTCTTAAACTTCCCATTAGGCAGAAATAATAGATTCATGCGCCGGTTCCTGGC

CTCGAAGCAGAGCCTCATCTATTCAGgagagaATGAGAGGTGATTGACGTCACAACTTCTGCACTTGGTTGAC

TCCGCTGTCTCAAAAGCCTCCTGCAGAAGTGAGTAATCAGAGACTAGAAGGTTGTCGTCCATAATGGTGGTTA

TTAGGATTTTAGTTGTTGGCTACCTTATCTCCTCAGATATCAACGTCCTTTCTTCATAGATTGATCTCAAATC

CCTTTCTTCTCAGATTGATCTCGAAGCCCTTTcaacaacaaGTCACAGATCTCGCAACTATTTCTTCTCATTA

GATCTCGTCAttcttcttcCTGTGATCATAGCTTATTTGATCACtttttCTCTCtattattattattatTTGA

AGTATTACGGaaaaaTAGTTTCTGCAACTGGTAAGAAACCCTTTTCTTAGTCTATCaaaaaaTAATTTTGATC

TGTCTTAATTGTATTCTAACTTGTAATGCAATAACCATAGATGATTTACTTTAAGGATTTTCTTCGAGGATTT

TCTAATTACTTGTAATAAGGTTTTGCTTCGAATGTTTTCAGAAGATGAtctctcTGCTTTGAGGGTTTGAAAG

ATTGATTATTTTACACTAAGAATTTGAAGGATAATTACTTGCAATGAGGGTTTCAATCATTAGAAGTTAAGAT

CATTGCTCAACTGGGTTGCCTAAACTAAAGTCTTATGCTACAAAGAG

E-84

CACAACACAAACCCTAGCCCTCTCACGCACACGCCACATATGCACGCTTCTTGACAGCGCACACCCACCTCCA

TCTTGTGTATCTGTGTACCCTCCCGATTTTCTCAGCAGAATCTATGTATTCGTATCACTACTAACCGTTTTCt

ttttCTTTCTTGTcacacaAACAAACAACCATACAAACATacacacAGTTCAGACATacaacaacaAGAAACT

TATACAACAATCActctctACAATTCATTCACAGCTAAGCTTTGTTGGtttttGATTATAACCAAATTtctct

ctctctatatatatttttGCTAATTTATTATAATTGGGAGAGGAGAATAATTCCACATGTACAAAAGGAGTCT

GGTGGCAGATTAGTGGCTCCGCGATCCCTCTTATCCACGTCGGCTTCCACCTTGGATTTCGATTCAATTCACA

GATTTCTTCTGTAACTTCTATAACAACTGTGTTTTGAGTTCCAAGTTCAGGAATTGTGTATGAACGGTTGGAT

TTGGTCATGGACAATGATGAGACAAATACACAAACAAAGCAATCaaaaaaTGTTGCTTCACAGGCCCAGGAGC

AAGCTCCTGTAAATTCTGCTCCAATGGCATACCCAGATTGGGCTTCAACGTTTAAGGCATATTATAACACAGG

aaaaaCACCACTTCATCCTGGAAGCTATTATACTTCTGCAGTTGCATCTAGTCCGCAGGCACACCCCTACATG

TGGCCACAGCACATGATTTCTCCATATGGTACACCACCTCCAACATATGTACCTATGTATTCACATGGAGCCA

TGTACACTCATGCAGCTATGCCTTCTGGTCCACATCCATATGGCTCATT

E-88

GATCAGTGGTGGCGGTGGCGGAGGATCTAGTAAGTGGGCGtttttCAACAAACTGGATGAACTCATCGGCCCA

TCGCGGAAGCATCAGCAGCATCCGCATCATCAACCACCGCCAGCGCCGCCTATGCCGAAGCTACCGACTCCTT

CACCTCCGATGAAGGCAGTTCCCATTAAGCACCGCGCCCACATGGAGGAAGTTGTGGTGACAAATCCGCAGCC

GCCTCAGCATCTGCATCATCACCTCCAATCAATGGTGATGCCCGTGGCGGTGTCGCTACCGCTGTCCGCACCA

ATGAATTTGATGATTCCAAACACTCGCATGCCGCCGCATCTGCATGTGACCTTGCCTGTCCCCACGGCCGTTG

CTGCTCCGAACTCGGCGTCCTCCAAGTCCGATGAGACGCCCGACACCACCGACAGTTACCCCAACGGCGGTGC

CATTAACGGAGGCAGCGGGGTGAAGAGTTCGAGtttttGCaaaaaGCGTAAACGCGAGGACACCGACACGGAG

GAATCGCCGCTGAAGGATCTGACGCGGGCGATAATGAAGTTTGGGGAGATTTATGAGAGGGTTGAGAGCTCGA

AGATGCAACAGCTGATTGATCTGGAAAAGCAACGGATGGTGTTCTTGAAGGATCTGGAGATTCAGCGGATGGA

CTATTTCATGCATGCGCACGTTGAGATGACCAAGCTCAAGCAAGAACGACGGCATCAACAACGCAGTGATGAG

CAGCGGGAGCacaacaacaacaacaacaACGGCAACAATGGCAACCACAATGATAATACTGAGCACTACTTGT

GACACCCATCAAAGGCGGATTCTTTATCACCACTGATCATCGCTTCAGAAGCCAGAGCA
