# Supplementary material for: Development of novel EST‐SSR markers for Ephedra sinica (Ephedraceae) by transcriptome database mining
Source: Appl Plant Sci. 2019 Jan 16;7(1):e01212. doi: 10.1002/aps3.1212 (PMC6342172; doi:10.1002/aps3.1212)
Supplement: Supplementary file 2 — APPENDIX S2. Polymorphic microsatellite target sequences from microsatellite marker development in Ephedra sinica. [file APS3-7-e01212-s002.docx]

APPENDIX S2. Polymorphic microsatellite target sequences from microsatellite marker development in *Ephedra sinica.*

>E-2

gagagaAGGCAAGTGTCATGGCAATGATGATAACAATAACAATGATGATAATGGTTGCAGCAGTAACTGCGCATCCATTCTCACgaggaggaggaggaggagGCAGAGGCGGTGGTCTCAGCCTCGGTTACTATGCAAGGACATGCCCCAAGGCGATGGATGTGATAAGACAAGGCGTCGAAGAGGCGATGCAGAAAGAGACGAGGATGGGAGCTTCGTTGCTCAGGCTTAGCTTCCACGATTGCTTCGTGCAGGGCTGTGACGCGTCTTTGCTCCTCGACGACAACTCCGCCAAGGGAATTGTCTCCGAGAAGAACTCCAATCCCAACAAGAACTCCGCCAGGGGATTCCAAGTCCTCGACACCATCAAGTCCCGCCTAGAACAAGCATGCCCCGCCACCGTGTCTTGCTCTGATATCTTGGCCGTTGCTGCCCGTGACGCCGTAGTGCTGAGTGGAGGTCTAGGATGGGATGTGCCGTTGGGAAGGAGGGACTCCAAGACCGCGAGCCTGAGCGGGTCGaacaacaacATACCGCCACCCAACTCGACTCTTCAGACACTGGTATCAAGGTTCAAAGCGCAGGGGCTGAACGAGGTGGATCTGGTTGCACTGTCCGGGAGCCACACCATTGGGCAGTCTCGGTGCACATCGTTCAGACAACGAGTGTACAATCGCACCTCGTACATTCAACAGTCGTTCTACCAGAAGCTCACCTCCAACTGCCCTCTCAGCGGAGGGGACAACAGActctctCCCTTGGACTTCCAGACTCCCACCAGATTCGACGGTTCCTACTTCACCAACCTTCTCAAAGGCATGGGCGTTCTCCACTCCGACGAGGTGCTTTCTACCGGTGACG

>E-18

GAGGcaacaacaacaaTATAATACCTTTGTGTTGACAGGaaaaaaaaaaNTCCAAACCaaaaaaaaTCTAGtttttGTACTGTACGaaaaaaaaTTAAATCTGtttttAGTATCAGAAGTTTTAATCCAGAAATTTTAGTTATGGCGATGAACCAGCAGAATagcagcagcTTGTACAACAATTACCAGCAGGCCTTCTGGAGATTCGACGAGCAGCTCCGTATGGAGCACGCCGGACTGAATAACAACAGTAACAGCAATATGAACACGAACAATCACGTGAATACTGTTGTGAGCAGTAAGTGGAACTTCACGCCCAGTAACGCCGTTCCTTTGTCCGCGTTTGCCAACATGAATCTGTTGGACAATGCAAACGCAAACCCTAAGTTGGTGaacaacaacTCCAAGACCGAGGCGAGGCGTAACAGCTACGCGGATCACAAGCCCAGCAACACCGCCGGGCGTGGCAAGTCGAAGCAGAAGGCTGACAAGAATCATAATGTTGACTGGAAGCACTCCGTAAATGAGAGCAATGAGAAGGTCAATGTCAATAGCCAGTGCAATGACAAGAATAGCaataataataataataatAACAATGGGAGCTTGGATAAGAGATTCAAGACGCTGCCTGCTACGGAGTCTCTTCCCAGGACTGAGATTCTTGGAGGTTATATCTTTGTCTGCAATAACGACACTATGCAGGATGATCTCAAACGCCAGCTCTTCGGTTTACCTCAGCGCTATCGAGATTCTGTTCGTGCGATAACGCCTGGTCTGCCCTTGTTTCTTTATAATTACACTacacacCAGCTGCATGGAAtttttGAGGCATCTAGTTTTG

>E-20

ACACCAATCCCTGAAGAGGCGCCAGCCCTGCCAAAAGACATCGTCCTCTGCGCCATTTCACAGGaaaaaGTATCGAAATTCCAAAATAAATCAAAACGCAATGCAACGCCGATAAATGTACAGACGATAATAATGCGATCGAAGGTATCAGGCCGAGATCCTAAGCGAGCCGTATGCGGTGTGGTGAGTCGCACGTACGGTTAGAGTTATGACATTATTTCCAGTTACGCGATTGCAGGGGTCGATGCTGTTTATCGGTATGGTGTCATCGCAGGAATGGTGCCAACTACTCTCGATTTATCCTACCATGGTTTCAATGCTGgtgtgtTTTCCAGGTACCTCAGACTGTGGTAATCCgggggAACATTGAGCGTATGCGTTGATCGCGTAGATTCTTGCAGGTTTGATGCCATTCCTCAGACTGGTATTATCGCTTTCCTTTGTTCCATGATCTATGGTTCTACGGGAGGTACCCAATTCGATCTATTAGCCGAGATGTTGACCGGAGATGCAACTACAAGTTTCGGTGCTCTTCGATCCGGAGGTATTTGGATGGGTATTCTATGTCTATCGGTAGGCtttttATTCAAGATCACAGCAGTTCCTTTTCGGGCGGGTGCATCGATTCGGATTAGGTGGAAAGCAAGCGGTGGCCAACTCTTACCATTTCGTGACATACGCGAGGTATCAGAATGTttcttcttcttcttcGTGCACGGATTCAGCAGAAGTACGAGCAGCTGCAATTGTGCTCAACAGGTACATGATCGGGTTGGATGGACAATCGCATGTTCGCCTAAGGTAACGTGAATGCTCGAGGTAGACGTAGACAGATAGCACGAAATACGCAGAAGTGGGACTGTTGGAACCTCACTCGAATCGTAATGGCGCATGGGAAGCGTAAGAAGCATGGTTGATTCGTACCCCTCGTACCAGCATCCAAGTAAAACTGCAAGGATCGGTAGGTACGGTAATTGCGCATGGTTCTT

>E-33

CTCCAAAGTACTGCACTTGATGATGTCTGTAGCGGCAGCTCCAAAGCCTCTGCATTTTCCATCATCAAATGACCTAAGAAATCAGTatcatcatcatcatcatcATAGATTCAGTGGGATTACTATACTAAAATCGCCATTATCAAAGTCTTTTCCGTCCTTCAAATCTTACCTGCTTTCCAACCGCGCTAAAGTGATGGCTGCCATTGGAGCAACTCCAACACAAGCCAACACTTCTGCCACTTTAGGTCACCAACAAACATCATCGGACTCCAAACAAGCGTTGATATCATTATCAGACAAACAGGATTTGGATGTCCTTGCTAAGGGACTTTCTGAATTGGGTTACACTATTGTTTCAACTGGTGGAACAGCATTGGCAATTGAAAGAATgggggTTCCCGTTACTAAAGTAGAGGATTTAACAGGGTTTCCGGAAATGTTGGATGGCCGTGTGAAAACTTTGCATCCTGCTGTTCATGGAGGGATTCTTGCAAGACGAGATCAAcatcatcatATGGAGGATCTAGAGAAGCATAAAATCAAGACATTTGATTTGGTGGTTGTAAATCTCTATCCATTCTATGAGAAGGTTTCCTCTTCTACCAAAGTTTCTTTTGAAGATGGAATTGAAAACATTGATATTGGTGGACCAACAATGATAAGAGCAGCTGCAAAGAACCATAAAGATGTTCTTGTAGTGGTTGATAGTGCTGACTATCCTTCTATTTTGAAATACTTACAAGgagagaTAAACATGGACGAAGAATCTTTTCGCAGAAAGCTTTGCCTGGAAGGCTTATCAGC

>E-35

CTTGTCGGCGCGGCTTCCAGACCTTGGATATTTCATACCCAATAACTGTTTtctctcCGCTGCCGATTTGCAGAAACTTAGGGCAATGCGAAACAAGGAATTGATCGGTGTTTCAGATGCGATTCAGAAGGCATGAGATTTCTAGGGTTTTagaagaagaagaagaagaGGAGAGTATCGTGAGGTGAAGAGAAGGGAGACATGTACAAGGACAGGGGTTCGAAATCAGAGCTGGGGAGCCTGAGGGATCAGACGGATCGCAAGAGGATCAACGATGCCCTCGACAAACATCTCCATCTCGACAAATCGTCACCGTCCACTTCCAACAACACCAATGCCAACGCCAAGGGCGCCGCGGTCCTCAACGGCCGCTTCTCCCTTCCTTCCACATCCTCTGCCGCACGCCGCATCAATGACAAGGcccccTCCTACGCCGAACCCGTAGAGGACTCTGAAACCGACAGCGAGGAATCAGATGTGAGTGGCTCAGATGGAGATGACACTTCTTGGATCTCGTGGTTTTGCAATTTGAAAGGAAATGAGtttttttGCGAGGTTGACGATGAGTATATTCAGGATGATTTTAATCTTTGTGGACTTAGCAGCCAAGTTCCTTATTATGATTATGCTCTTGACTTGATTCTAGACGTCGAGTCATCTAATGATGACATTTTGACCGAGGAACAAAATGAACTAGTGGAGTCAGCTGCAGAAATGTTATATGGCTTAATCCATGTTAGATACATTTTAACAAGTAAAGGGATGAATGCTATGCTTGAAAAGTATAAGAGCACAGACTTTGGCAGATGCCCAAGAGTGTACTGTTCCGGACAACCCTGCCTTCCAGTTGGTCAATCTGACATTCCGCGAACCAGTACAGTA

>E-49

GGAGAATGGAGTGTTGCAGATTCCAATAAATGGGACTCCTTGAGGCGCTTTATTCAGTGGTGCAATCGCTTACCACAGGCCAAGAAGGTCGCGACAAGCATAGCTCCACTTCCTCTTCCAGTTCCATGGCGGTGggaggaggaggaggaGAGATATCGAGAAAGCTGGAGGACAAATTGAATGGGTATTTCGATCTTGCGAAGAAGGAAATTGACAGAGCCGTCCGAGCTGAAGAATGGGGTCTTCCATCCGAGGCTATTCCTCATTATAAAACAGCTCACCGCATCCTTTCTGAAGGCCTACAGGTTCCTCTCCCCTCTTCCAGTTGTTCAAAGGATATCAAGGATTGCCAAGCTAAGTTATTGAAGTGGCAAGGGAATGTTTATGATAGACTACAATACCTAGAATTCCAAACAGGTCACTCGTCAAACCAGAGTGCATCTAATCAGGCGCTGACACAGCCACCAATGCGAGCAAAACAATCTTTTCGTAGCAATTCCCAAAAGCAAAGTTCCATTGTTTCAGGTCACTCGTCAAACCAGGCGCTGTCACAACCACCAATGCGAGCAAAACAACCTCTTCATAGCAATTCCCAAAAGCAAAGAtttttGAACACCAGTaaaaaTCTTTCTAAAACAAGTGTGCCTGTAAGCTCTTCCGCAACTTCTCATGTAAACAAGGTTGATCTTAAAGGGATCGATTCTCAGCATATGCAAATAATAGAAAGTACAATTGTTGATAAAAGTCCAGCAGTTAAATGGGATGATATAGCTGGGCTTACAAAAGCAAAACAAGCTTTAAGAGAAATGGTCAT

>E-58

TCTGCTTTTCTTTCAAGATCCCCAATCTAATTAACTTTTCTTGTCTTGACAATGAACCTCAAGGTGTAGACAGGGGATCCATGAATCCGAAGGTGTAGAATCTAATTAATTTCTCTTGTCTTGGACAATGAAGCTGAAGGTGTAGACAGGGATGACGAGGTTCATTGGCACGGACGAGATTCTCGGGGCACGCGCTGCCTCCGCTCTGTCGAGAAGAACCGACGAGGGCAGCGCTCGCCGACTACGTGAATTaaaaaaGCAGCTCAGCCGGCTAGTTCATGTCGTCACGgatgatgatgatgatGACCATAATTTCAGAATCGGAGTCTTGGATCAGATTTCCAacacacTCAACGCGCTCAAGGACCTCAAGTTCCACCCTTCACCGACACCGCCTCACACTCACGCCCACAGCTCCATTGCCGTGCCTGACCATTTCCTGTGCCCCATCTCCTCAGAACTCATGTCCGACCCCGTCATCATCGCTTCTGGCCAGACATACGACAGAGCATACATAGAAGCGTGGTTGGCAGCGGGCAACAGAACGTGCCCAAAGACGAAGCAAGTACTGacacacacTATGCTTACACCCAACATTCTAGTGCGAAGAATGATACAAGACTGGTGCGAGTCGGAGGGAGTTGAATCCCCTAGATGCTCCTCCAAGAATGAGACCACCGTGTCCGAGTCGGTGTCCGTGTCGGGAATAACGTTGGAagagagAAGGTACGTGGCGGTGTTGTTGgagagaATAATATTAGGTAACAATGGCGGAACAGGGTCATGTGATGCGGTTAAAGAGCTGAGAGCGCTGA

>E-59

GTTTTGGTAACAGAGATGAAGATGGATTCAGAAGTCCTATGAGAGGGGATCCAAGATCTGGAAGGAGAAGCAGTAGGTTCGATTCTGATGATTTGGATGAAGATGACATGGACGATTTCGGAAGTGGAAGGAGGGGTTCAGGGTTTGGAAGAGGAAgaggaggaggaggaggaggaggaggagGACGAGGAAGTAGAATGGGAGATTCTGAAGACATGGATGATGACATGGGCTTTGGAAGGTCAGGAAGAAATAgaggaggagGTGGTGATTTTAGAGAAGAATTTGGTGATAGGAATGGAAATAGAAATTCAGGGTTTTCAAGAAGACCAGGACGTGGCCAAAGGGAGATGTACTATTCATCCTTTGCAGGTTCACAGGGATGCCTAGAGACATTGGAATCTTGACTGATGATACATGTTGAGGCTCTGGTTGACTGGCGCAGAGCTTGACACTTGAAGGCCAAATTTGAAGAATCGTCGCTGGTCTAATATTTTCTGAGTCCtttttCCTTGaaaaaCCTTGTCACCAACTAGCCAGGTTTGTGTTTCAGTAGaaaaaTCtttttCTTTTATTTCTAAAATGCAAAGTTTTATAGGAGAAGGCCGATTTAGTTGTTTAGGCAATTTCAAGTTCTCTTGTTTGAGATGCTTTGTTACTTTAACATCCACCACTAATTTAATGGTGATCCT

>E-62

GTAGGAAGAGGTGTCCAagagagGGGTGTGCGTATCATTTGCCaaaaaGGAACTAATGAACACTACGACTACACAGTTTAATGTCTTAGTCACGCCGTTCTTACAATCCATCTGCAAAATAAATGCAGCCAGTGTACCATTGTTTCCCAAGAACATACGATTCCTTGTCTGAAAGCTTGTTTTGTAGCCTCCCTTCTGTAGCAACACTAGCTGTATTTTCTCCAAACGCAATCCCCTTTATTTTGActctctTTTATCCAGTCCCCACATCCCTCTTTATATCACAATAATGTCTTTTATGCCTTTGGTCTTTGTTAGTTAAACTCATTCAGGTTGAgtgtgtGAGAAGAATTGTGTCAAAGAGTTTGGAAATTGTTGTTAAAGATGAATAGAAGCTGGCTGGGTGTTGTGGATACTATCTACgaagaagaaGGCCaagaagaagaagaagAGTCACACCATTCTTGCTCAGATTCAGACTCTAGTAGTTGTGTTGATGCTAGGCAGAAAGTCTCTTCGCAGTCATTACATACTTCATGGATTGTCACTAAGGATGAGGACACAATCACATCAGACAGAACCAGCCAAGGAGATgaagaagaaGACCATGATCCAtctctcCAACAAATCATTGCCTCTTGGTCTGAAGCAACCGGTATCACAGCAAGTATCACAATTAGAATTGGAGATCGTAtttttGTAATGCATAGGTTTCCACTTGTGTCAAGGAGTG

>E-71

aaaaaGCAATGGAAGAATTCGGAATGATGAAGTTCTGGTCCCTTATGGGAGTTCTTGCAGTTCTGCAGAATCTGGTGAACCACCCTTTGGTGGCATATTTGTTTAGATGGCTGGAGACATGGAGGGGCAAGAACAACTCCTTTCACtttttCAGAATTCCCCAGTTCGGGGACACCGGCAGCACCGCCACCACCGTCATGGACATGGACCAAGGCAGCTATTACAACGGATTCCAAGAGAATGAGATGTATAGGATGGCAATGACCTACGTGGGCACCTTGAAGGGAGTCGCCGACACAGAGTATGCAAATGTGTACGTCTCCAGGCAGACCAACCGTAATGAAACCAAGATGAAGAGGTTCCTTTGTTTGGAGAACGGAGAAAGCGTGCAAGACGAGTTTATGGGTGTTGAGGTGTGGTGGACTCATGATCATAAGTCCCCTCGGCCGTCAAAACAGCAATCATATCGGGGCATGGACGATGGCAACACcaacaacaaCGacaacaacaacaacaacaCAAAGAGTTTCGTGCTCAAGATGGCGAAGAAGGACAAGGAGGTGGTTCTGGGAGAGTATCTGGACCATGTGGTGAGCGTGGCTATTGAGGTGGAGAGGAAGAGGACTCAGGTGATGTTGTACAGCAACAGAGGCGGAGGATGGTCCGCACCCGTTCCATTTCGGCATCCTTCCACGTTCGACACCATCGCCT

>E-83

CTAGAACTACCTCGGTGGAGTCCAGAAAGAGAAGCTCAATAAATCTCGGATATGGATGATTTAAGAGCTCTTAAGGTGATAGGATGCGGAGGCATGGGCACAGTGATATTGGTGGaaaaaGAAGGCTCCgagagaCCCTTGGCACTAAAGGCCATGAACAAGTCCGTCATCTCagagagagTTGACGGCCTTGAAAGAGCCGAACTagagagGAACATtctctcAAAGCTTCACCATCCCTTTCTTCCGGAGCTTCTAAGCTCCATAGAAACGGaaaaaaCGGTGGGCtttttGATGGAGTACTGTCCGGGAGGAGACTTGCActctctCCGCCTCAAGCAAACCgagagaGCCTTTTCAGAGTCCATCATACGGTTCTACGCTGCGGAGATTGTCTTAGCATTGGAGTACTTGCACAAGATGGGTATTGTCTACAGAGATTTGAAGCCCGAGAACGTTCTCATTCAATCCGACGGTCATGTCATGCTCACCGACTTCGAtctctcCGCCTTCATCACTCCTCCTGCTCCGTATCAAGAAGAGCGACAAAGCTTGGATGATGTCAAGAGGAAGCCATCCAAGAGTAGTGTAGTACGAAGAATGCTGAGAAGatcatcatcatcatcttcttcttcTCAGAAGGCAGAGAAGTGTAGCAATACATCACCGTCATCGGCACGGGTGACGCCGGCGAAGGAGAGCAATGAGAAGTCGCACTCCTTCGTGGGCACCGAGGAGTACGTGTCTCCGGAAATGGTGAGCGGCAAGGACCACGACTTCAGCGTGGATTGGTGGGCACTCGGTGTTTTACTCTACGAAATGGTATATGGaaaaaCGCCATTCAAA
